# The Throat Hospital, Golden Square

**Published:** 1895-09-07

**Authors:** 


### EDITOR'S LETTER-BOX.

[Our correspondents are reminded that proxility is a great bar to publication, and that brevity of style and conciseness of statement greatly facilitate early insertion.]

### THE THROAT HOSPITAL, GOLDEN SQUARE.

DR. WOLFENDEN writes: With reference to your annotation anent the affairs of the Throat Hospital, Golden Square, and the statement therein made that it is the duty of the committee, or of the three gentlemen who have made the announcement of their resignations with such a grand flourish of trumpets, to justify themselves to the public, I take the opportunity of saying, as one of the recently-elected members of committee of the above hospital, that if the subscribers to the institution desire any inquiry into the circumstances which led to the resignations in question, I, for one, shall be only too glad to assist such an investigation. The present committee has nothing to fear from such a course. The gentlemen who were elected to the committee in May last, of whom I was one, have accepted the position with the fullest intention of discharging their duty to the public, of preventing the hospital from again being made an area for the furtherance of private interests, and of bringing the government of the institution into line with that of older and esteemed public charities. The violence with which our elections have been assailed by a small section of individuals is explicable easily enough. It is, however, not for the committee to justify themselves, but for the three gentlemen in question, if their resignations are regarded by the subscribers as of sufficient public importance. The only one appearing to be so is that of the Duke of Sutherland, and the first question which should be answered in this instance, is how, after having given an undertaking that if he resigned he would not, in the interests of the institution, make it a matter of public notoriety, his Grace allowed himself to be a party to the issue of a manifesto of the most public character within a day or two after the acceptance of his resignation by the committee. The retirement of the late chairman was explicable by the fact that an old member of the committee, whom he opposed for the position, was unanimously elected. The third resignation appears to have been an entirely personal matter of no importance. The present committee is quite in accord, and quite alive to the necessity of so altering the rules and constitution of the hospital that it can never again become the area for the advancement of private aims, and this will be the first work of the new committee. I write this at a distance, a copy of your paper having been forwarded to me here only yesterday.

